# Auto-antibodies against apolipoprotein A-1 block cancer cells proliferation and induce apoptosis

**DOI:** 10.18632/oncotarget.27814

**Published:** 2020-11-17

**Authors:** Nathalie Satta, Rémy Weppe, Sabrina Pagano, Miguel Frias, Catherine Juillard, Nicolas Vuilleumier

**Affiliations:** ^1^Division of Laboratory Medicine, Department of Diagnostic, Geneva University Hospitals, Geneva, Switzerland; ^2^Department of Medicine, Medical Faculty, Geneva University, Geneva, Switzerland

**Keywords:** anti-apoA-1 antibodies, apoptose, cell proliferation, oxidative stress

## Abstract

Auto-antibodies against apoA-1 (anti-apoA-1 IgGs) have been identified as important actors of atherosclerosis development through pro-inflammatory and pro-atherogenic properties and to also induce apoptosis in tumoral neuronal and lymphocyte derived cell lines through unknown mechanisms. The purpose of this study was to explore the cellular pathways involved in tumoral cell survival modulated by anti-apoA-1 antibodies. We observed that anti-apoA-1 antibodies induce growth arrest (in G2/M phase) and cell apoptosis through caspase 3 activation, accompanied by a selective p53 phosphorylation on serine 15. RNA sequencing indicated that anti-apoA-1 IgGs affect the expression of more than 950 genes belonging to five major groups of genes and respectively involved in i) cell proliferation inhibition, ii) p53 stabilisation and regulation, iii) apoptosis regulation, iv) inflammation regulation, and v) oxidative stress.

In conclusion, anti-apoA-1 antibodies seem to have a role in blocking tumoral cell proliferation and survival, by activating a major tumor suppressor protein and by modulating the inflammatory and oxidative stress response. Further investigations are needed to explore a possible anti-cancer therapeutic approach of these antibodies in very specific and circumscribed conditions.

## INTRODUCTION

Autoantibodies are considered as the hallmark of B cell-dysregulation and are predominantly retrieved in autoimmune and neoplasic diseases, as well as in various paraneoplasic syndromes [1–3]. Autoantibodies can be produced by B1 or B2 lymphocytes [4]. Those produced by B2 lymphocytes can be pathogenic through several mechanisms including FC gamma receptor activation, direct cytotoxicity, complement activation, innate immune receptor stimulation, or cross reactivity due to molecular mimicry [5, 6]. Most tumor autoantibodies reported so far have shown some potential for early detection, prognosis and response to therapies, but their role as modulators of tumoral cell survival remain elusive [2, 3]. In the field of cardiovascular diseases (CVD) sharing similar physiopathological pathways with cancer [7], several autoantibodies are involved as mediators of the disease [8]. Among these, autoantibodies directed against apolipoprotein A-1 (anti-apoA-1 IgGs), the main protein of high-density lipoprotein (HDL), have been shown to be actively implicated in the development of atherogenesis and atherosclerosic plaque vulnerability by activating macrophage-dependent inflammation, inducing foam cell formation, disturbing HDL-cholesterol homeostasis, and promoting a pro-arrhythmogenic effect [9–12]. Due to molecular mimicry between apoA-1 and the extra cellular parts of toll-like receptor 2 (TLR2) [9], anti-apoA-1 IgGs bind to TLR2 trough their Fab parts, inducing the constitution and activation of a TLR2/TLR4/CD14 heterotrimer complex followed by nuclear factor kappa B (NF-kB), activator protein 1 (AP-1), and tyrosine protein kinase Src dependent pathways activation [9, 13].

Lately, anti-apoA-1 antibodies were also found to bare dose-dependent cytotoxic properties on glioblastoma cells and lymphoma cells (but not on non-tumoral primary cells) independently of TLR2/TLR4/CD14 signalling [14, 15]. Therefore, we investigated the intracellular molecular mechanisms underlying anti-apoA-1 IgG-induced cytotoxicity on tumoral cells using various cancer cell lines, and compared them to non-tumoral cells. We expected by cross-checking information gathered from tumoral cell lineages from various genetic origins to identify a dedicated pathway triggered by anti-apoA-1 IgGs.

## RESULTS

### Cellular morphology changes induced by anti-apoA-1 IgGs

**Figure 1:**
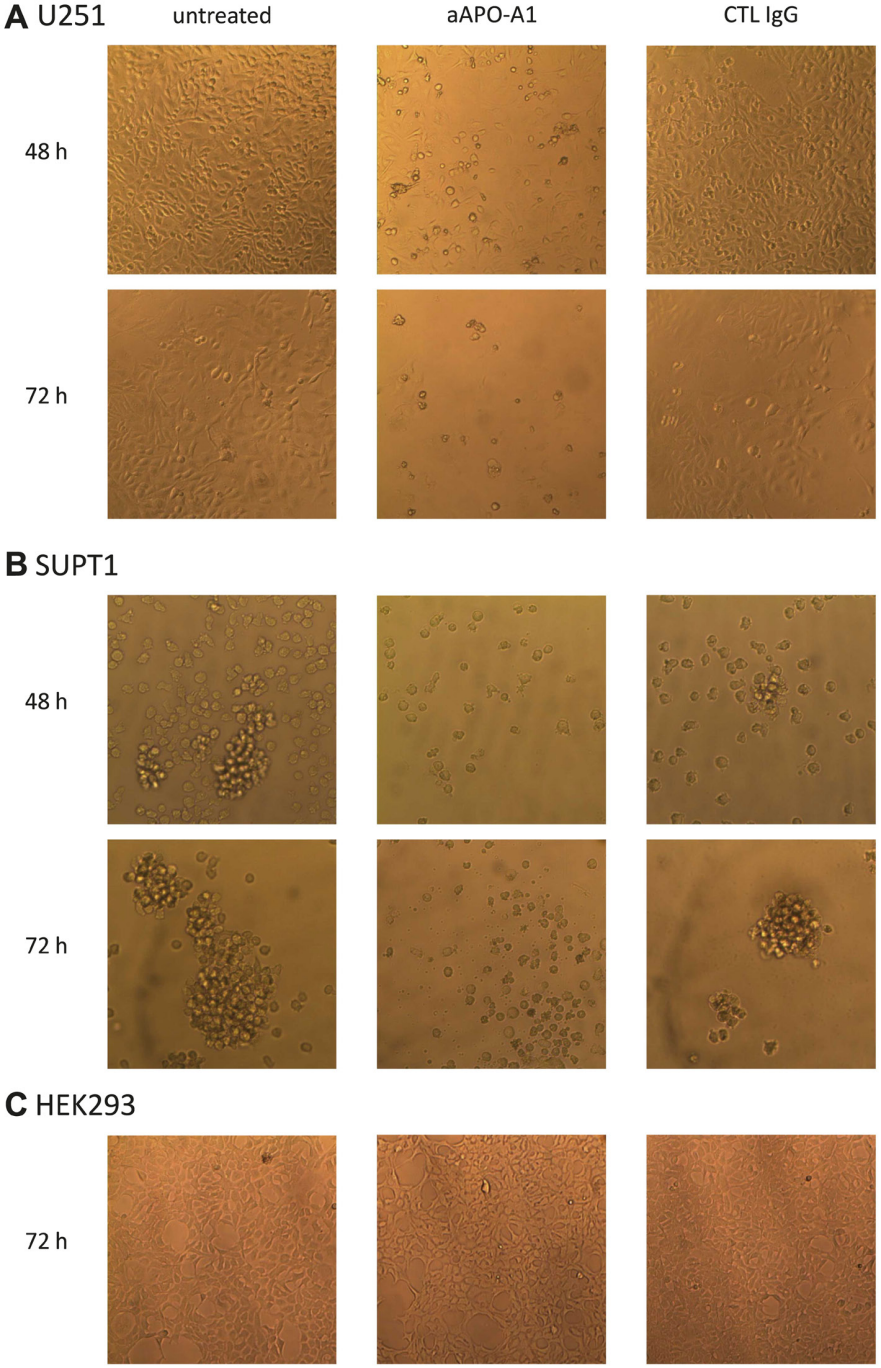
Morphology change of cells cultured in presence of anti-apoA-1 IgG or control isotype IgG. Pictures of U251 (**A**), SUPT1 (**B**) or HEK293 cells (**C**) in culture were taken at objective ×10 using a Axiovert 25 (Zeiss) microscope equipped with a CETI SI-3000 High-Definition Digital camera. Cells were incubated with goat polyclonal anti-apoA-1 IgG (aAPO-A1) or isotype CTL IgG (CTL IgG) at 150 μg/ml for 48 or 72 h.

Our investigation started based on the observation that the addition of anti-apoA-1 IgGs in the cell culture medium induced a change over time in cell morphology of cancer-derived cell lines from different genetic background (Hela, U251, SUPT1), but not primary cells (macrophages or HAEC) nor immortalized cell lines (HEK293), whereas the isotype control IgGs had no visible effect. As shown in Figure 1, the cancer-derived cells U251 (Figure 1A) and SUPT1 (Figure 1B), when treated with anti-apoA-1 IgGs for 48 and 72 h, presented the typical hallmarks of apoptosis: rounded and shrinked cells, detachment from the flask for U251, retraction of the nucleus with chromatin condensation, emission of apoptotic bodies. These apoptotic features were not observed in HEK293 (Figure 1C) cell culture.

### Anti-apoA-1 IgGs stimulate apoptosis and necrosis on tumoral cell lines through Caspase-3 activation and p53 phosphorylation

**Figure 2:**
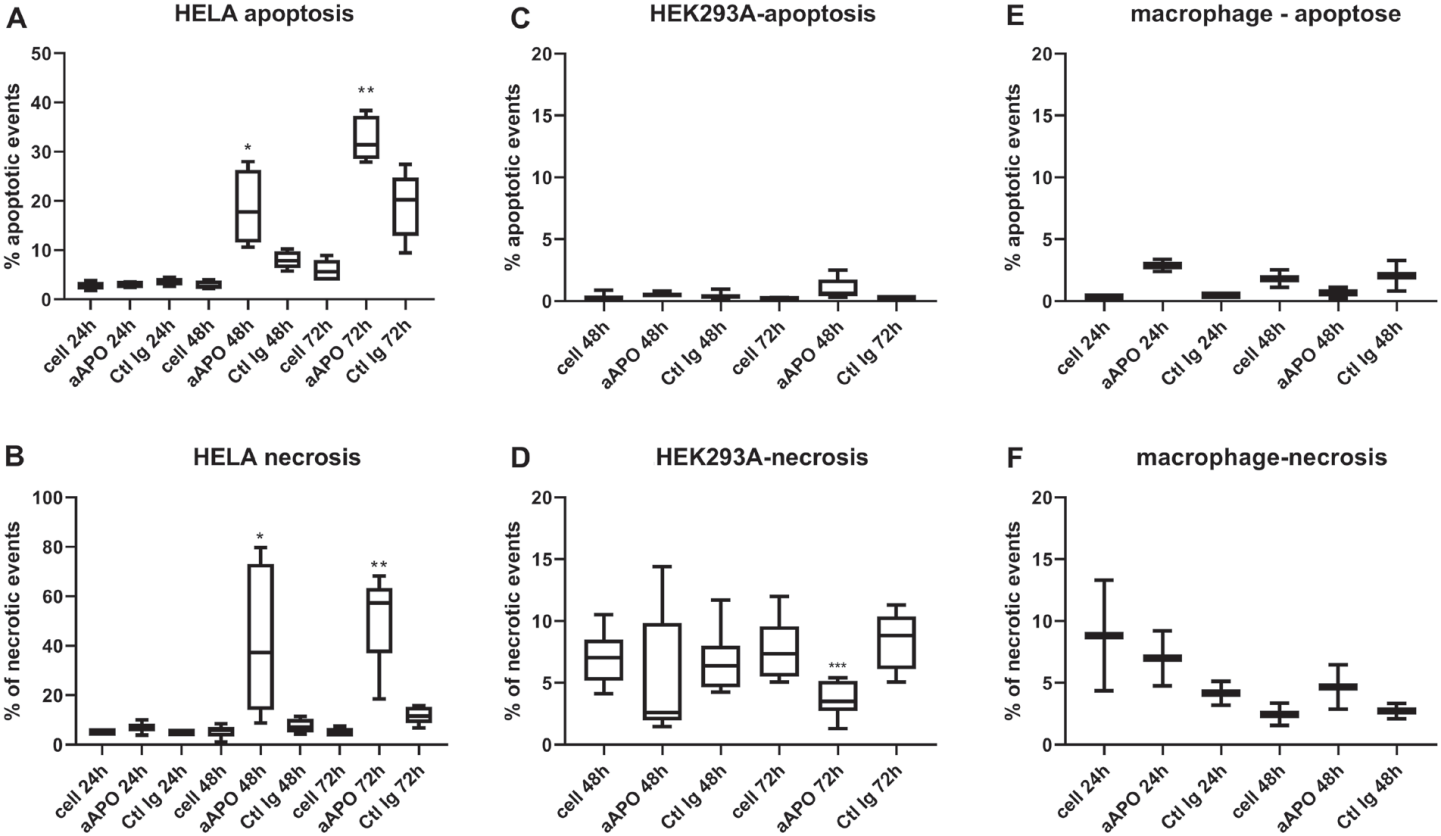
Apoptosis and necrosis quantification after incubation of Hela, HEK293 and macrophages with anti-apoA-1 IgGs. Data are expressed as % of apoptotic (**A**, **C**, **E**) (activated caspase 3 positive cells) or necrotic (**B**, **D**, **F**) events quantified by flow cytometry after 24, 48 or 72 h of cell incubation with polyclonal goat anti-apoA-1 or polyclonal goat control antibodies (150 μg/ml). Data are presented in Box and Whisker graph with median, IQR and range. Significant difference between anti-apoA-1 and control IgGs was assessed by Mann Whitney test on 5 to 8 independent experiments (^*^ *p* < 0.05; ^****^ *p* < 0.001), only 2 experiments were performed with macrophages.

**Figure 3:**
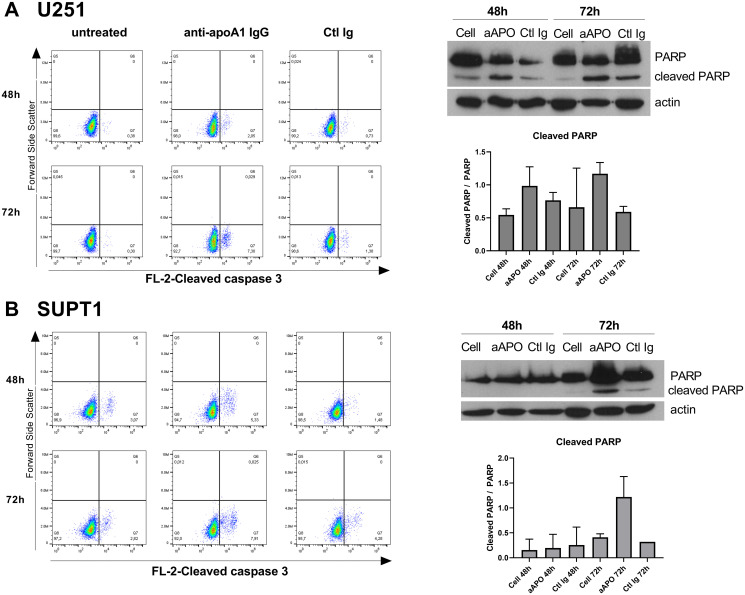
Anti-apoA-1 IgGs induced Caspase 3 and PARP cleavage in U251 (**A**) and SUPT1 (**B**). Dot plot analysis of cleaved caspase 3 according to cell treatment for 48 h or 72 h. The percentage of cleaved caspase 3 enriched cells is indicated in the Low-right panel. Western blot analysis of PARP cleavage after 48 and 72 h of U251 (A) and SUPT1 (B) cells treated with anti-apoA-1 IgGs or CTL IgGs at 150 μg/ml. Graphs present the mean+/–SD ratio of cleaved PARP over PARP, normalized to β-actin from 2 experiments.

Based upon our morphological evaluation suggesting that anti-apoA-1 IgGs could stimulate tumoral cell apoptosis, we measured the impact of anti-apoA-1 IgG treatment on caspase-3 activation, known to be a key driver of apoptosis [16]. As shown in Figure 2, anti-apoA-1 IgGs induced the activation of caspase 3 in Hela, another carcinoma derived-cell line but not in HEK293A or human primary macrophages (Figure 2A, 2C and 2E). Anti-apoA-1 IgGs also induced a significant necrotic effect on Hela (Figure 2B), which was not observed on HEK293A and macrophages (Figure 2D and 2F). Polyclonal goat control IgGs had no effect (Figure 2). Anti-apoA-1 IgG-dependent activation of caspase 3 was also attested in U251 and SUPT1 by an increased percentage of cells with cleaved caspase 3 and an increased level of cleaved PARP, one of the main caspase 3 substrate protein (Figure 3A and 3B).

**Figure 4:**
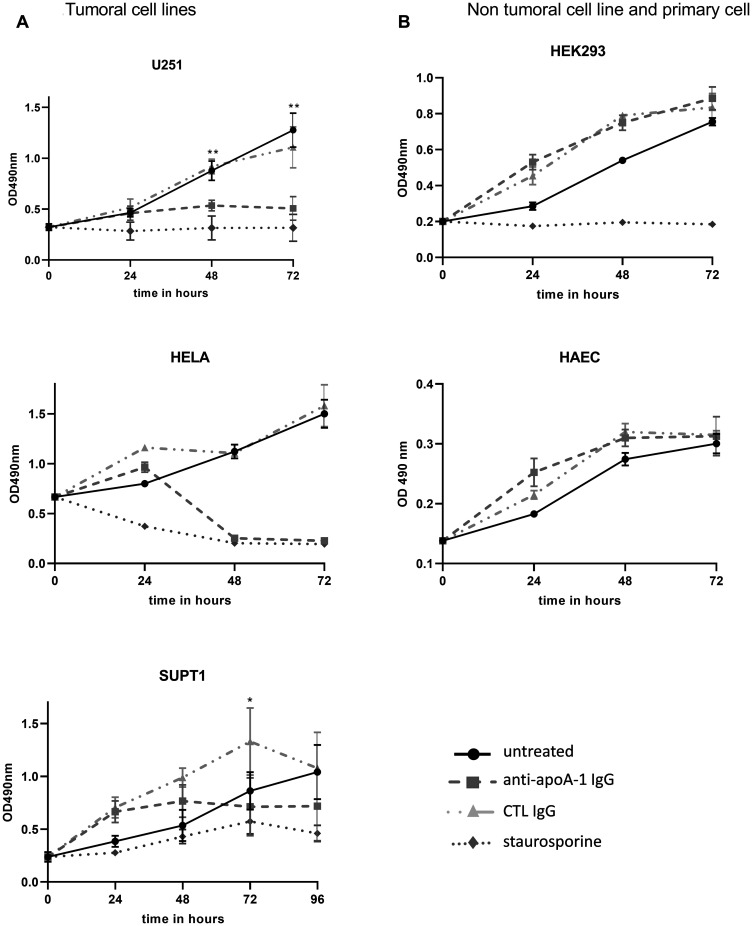
Effect of anti-ApoA-1 IgGs on cell proliferation. Cell proliferation and viability were quantified by MTT assay over 96 hours in tumoral cell lines (**A**) and non tumoral cell line and primary cell (**B**). Apoptosis inductor staurosporine was used at 1 μM and polyclonal goat anti-apoA-1 and polyclonal goat CTL IgGs at 150 μg/ml. Data are expressed as means + SD for 2 to 4 independent experiments. Significant differences between anti-apoA-1 and control IgGs, and untreated cells were assessed by Mann Whitney test (^*^ *p* < 0.05).

As shown in Figure 4, this pro-apoptotic effect was accompanied by an anti-apoA-1 IgG-induce tumoral cell proliferation inhibition. Anti-apoA-1 IgGs, but not control IgGs, induced a cell growth arrest after 24 h treatment followed by cell death in U251, Hela, and SUPT1, in the same range as for staurosporine, an apoptotic inducer (Figure 4A). By contrast, no proliferation inhibition was observed on the two non-tumoral cell-lines tested (Figure 4B).

**Figure 5:**
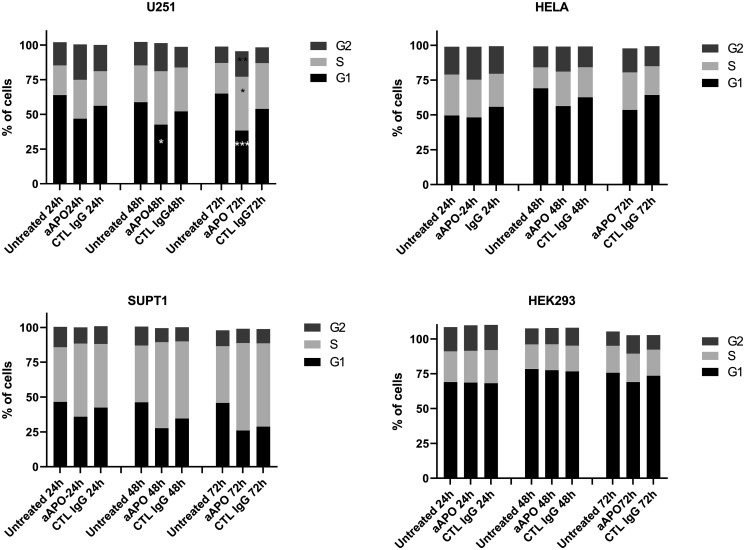
Modification of cell cycle phases according to cell treatment. Cell cycle profiles were determined by flow cytometry and percentage of cells in G1, S and G2 phases were calculated using Watson model. Data are expressed as mean of % of cells from 8 experiments for U251, 4 experiments for SUPT1 and Hela, 2 experiments for HEK293. Significant differences between anti-apoA-1 and control IgG, and untreated cells were assessed by Mann Whitney test (^*^ *p* < 0.05).

To further investigate the mechanism of growth arrest, cell cycle experiments were performed on U251, Hela, SUPT1 and HEK293A in presence of anti-apoA-1 or control IgGs at 150 ug/ml over 72 h of culture. Significant changes in the cell cycle profile were observed for U251, SUPT1 and at less extent for Hela treated with anti-apoA-1 IgGs (Figure 5). The proportion of cells in G1 phase significantly decreased and a concomitant increase in S and G2/M phase population was observed. This is reflecting a cell cycle arrest at the G2/M transition. No difference between conditions was observed to HEK293A cell cycle profile.

**Figure 6:**
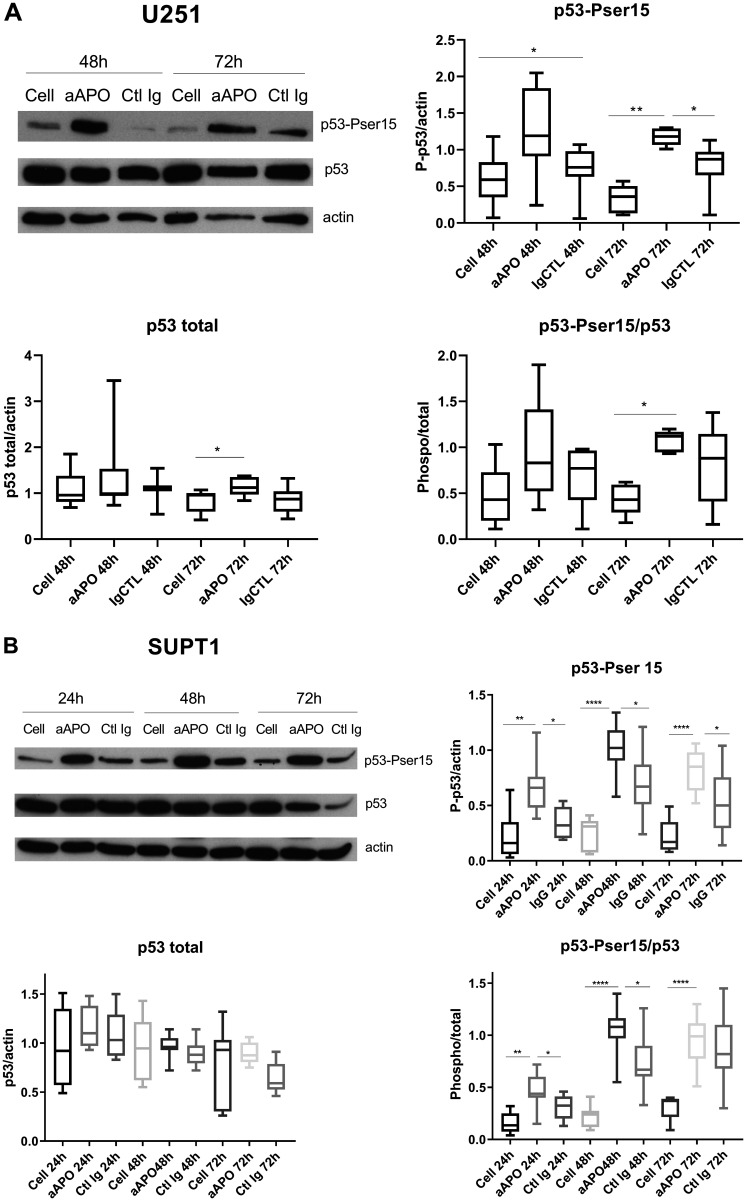
Anti-apoA-1 IgGs induced p53 phosphorylation on Serine 15. Western blot analysis of p53 total expression and phosphorylation on Ser 15 after 24, 48 and 72 h of U251 (**A**) and SUPT1 (**B**) cells treated with anti-apoA-1 IgG or CTL IgG at 150 μg/ml. Graphs present the expression of the p53 or phosphoSer15-p53 normalized to b-actin in Box and Whisker graph with median, IQR and range. Significant differences were assessed by Mann Whitney test on 6 independent experiments (^*^ *p* < 0.05).

**Figure 7:**
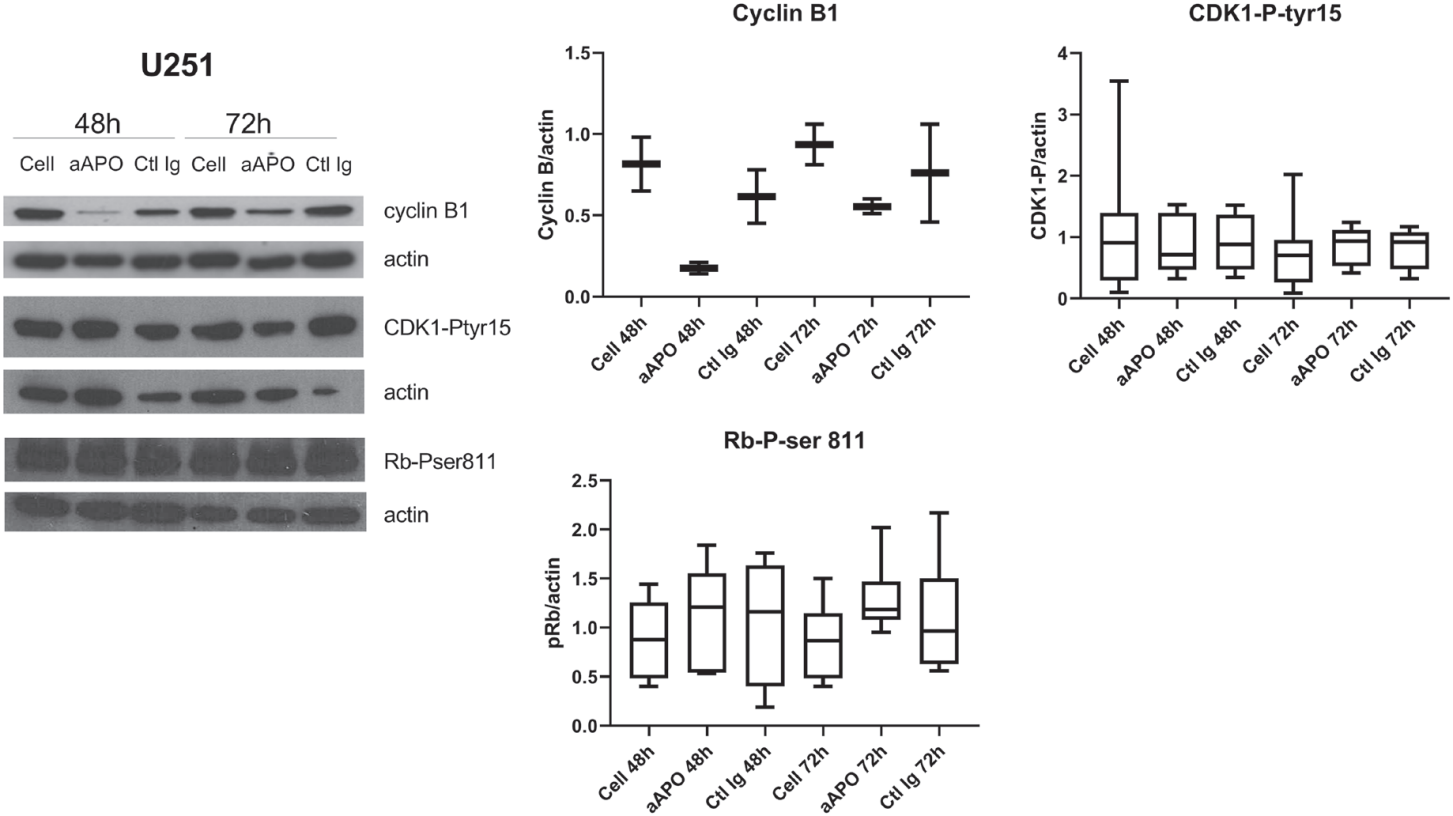
Anti-apo-A1 IgGs induced a down-expression of cyclin B1 in U251. Western blot analysis of cyclin B1 expression, CDK1 phosphorylation on Tyrosine 15 and Rb phosphorylation on Ser 807/811 in U251 after treatment with anti-apoA-1 IgGs or CTL IgGs at 150 μg/ml. Graphs represent the quantification of the target proteins normalized to b-actin from 2 (cyclin B1), 8 (CDK1-P-tyr15) or 6 (Rb-P-ser81) experiments in Box and Whisker graph with median, IQR and range.

These investigations were completed by western blot analyses where the levels of p53, a key tumor suppressor protein in vertebrates controlling cell proliferation by regulating G2/M transition and cell death according to survival or damage signals received by the cells [17], were evaluated in response to anti-apoA-1 IgG treatment. Its functional status being tightly controlled by several phosphorylation/dephosphorylation processes, the phosphorylation status of p53 was also evaluated in the different cell lines. As shown in Figure 6A and 6B, p53 was strongly and selectively phosphorylated on Ser15 in U251 and SUPT1 cells exposed to anti-apoA-1 IgGs, although no variation of the quantity of the protein was detected. No other p53 phosphorylation (e.g., Ser20 and Ser46) was observed (data not shown). With regards to HEK293, HAEC and macrophages, no phosphorylation of p53 was observed in any of the conditions tested. In accordance to the reported p53 repression induced by HPV16 E6 oncoprotein in Hela [18, 19], we did not detect any p53 expression in Hela cells (data not shown). Because p53 regulates the expression of GADD45 alpha which interacts with CDK1 to promote cyclin B1 degradation [20] leading to a cellular cycle arrest in the G2 phase [21], we investigated the level of cyclin B1 and the phosphorylation of CDK1 on tyrosine 15, in U251 treated with anti-apoA-1 IgGs by western blot analysis. A strong decrease of cyclin B1 expression was observed in cells treated with anti-apoA-1 IgGs at 48 and 72 h in comparison to untreated (Cell) or control IgG treated cells. No change in CKD1 phosphorylation on Tyrosine 15 was observed (Figure 7).

We also investigated the level of phosphorylation of the retinoblastoma tumor suppressor protein (Rb), another major oncogene, controlling G1/S phase cell cycle progression dependently on Cyclin D/CDK4/CDK6 [22]. As shown in Figure 7, no change in Rb phosphorylation on Ser811 was observed in any of the conditions and irrespective of the cell lines.

Finally, PUMA, NOXA and BAD, three pro-apoptotic proteins that are transcriptionaly up-regulated by p53 and participated in mitochondria-dependent apoptosis [23, 24], were also evaluated in response to anti-apoA-1 IgG treatment. None of these proteins were differentially expressed under anti-apoA-1 IgG treatment either in U251 nor in SUPT1 cells (data not shown).

### Impact of anti-apoA-1 IgGs on the U251 cell transcriptome

Because phosphorylated p53 on serine 15 induces the transcription of specific genes controling cell proliferation and/or apoptosis [25, 26], RNA sequencing analysis was performed to identify differentially expressed genes in U251 treated with anti-apoA-1 IgGs in comparison to untreated or Control IgG treated cells at 3 time points of culture, 24 h, 48 h and 72 h. We found 978, 2418 and 2646 significant genes specifically regulated by anti-apoA-1 IgGs at 24 h, 48 h and 72 h treatment respectively. To investigate whether in these lists of genes, some were involved in apoptosis or cell cycle control, we cross-mapped the gene lists with the pathway maps for apoptosis, cell cycle and DNA damage ontologies in Metacore. The corresponding genes are summarized in Supplementary Table 2. For a large majority of them, their expression was not found significantly modified at 24 h of treatment with anti-apoA-1 IgGs, but were significantly modulated at 48 and 72 h, which is in aggreement with our observations indicating a visible effect on anti-apoA-1 IgG-induced cell survival mostly after 48 h of treatment. Five major groups of genes were found to be modified by anti-apoA-1 IgG treatment.

The first gene group relates to those involved in cell cycle regulation and cell growth. All the genes known as inhibitors of cell proliferation but one (CAMKI) were up-regulated. Several of them are direct transcriptional targets of p53: 14.3.3 sigma, GADD45A, B, G, CDKN1C, CDKL2, PLK2, BGT4, PIG3 [27–29]. The 14.3.3 sigma, GADD45 alpha and CDKN1C proteins are inhibitors of CDK1, the serine/threonine protein kinase that forms active complex with cyclin B1 and controls the G2/M transition. BTG4 and RERG genes implicated in tumor growth repression [30, 31] were found to be the most strongly up-regulated genes by anti-apoA-1 IgGs (Supplementary Table 2).

The second group of genes found to be differentially expressed were those known to be implicated in p53 stabilisation and regulation. Among these, VRK2 and PLK3 (p53 stabilisation and activation) where overexpressed, whereas CDKN2AIP gene (p53 expression inhibitor [28]) was downregulated (Supplementary Table 2).

The third group relates to genes involved in apoptosis regulation, where we noted an upregulation of several pro-apoptotic genes, such as the TNFRSF, granzyme M, and RhoB, TP53AIP1 as well as a downregulation of the anti-apoptotic PAK1 gene. No transcripts of pro-apoptosis or anti-apoptosis BCL2-family proteins were found at the exception of NOXA that was mildly up-regulated at 72 h and cIAP-2 at 48 h (Supplementary Table 2).

The fourth group of genes concerned belongs to inflammation regulation, where several genes related to the familly of death receptors (TLR and TNF receptor super family) were modified, with an up-regulation of TLR6 and CD14 (the co-receptor of TLR2 and TLR4), NF-KB p100/p52, TRAF3 and the TNF-superfamily ligand 9 (TNFSF9, also known as CD137L) was found (Supplementary Table 2).

The last group of genes concerned was those associated to oxidative stress, where the exposure of anti-apoA-1 IgGs led to an important upregulation of the nitric oxide synthase (Supplementary Table 2).

## DISCUSSION

The most notable finding of this study was that anti-apoA-1 autoantibodies induced tumoral cell growth arrest in G2 and cell death through caspase 3 activation, accompanied by a selective p53 phosphorylation on serine 15, associated with cycline B1 degradation and the modulation expression of key genes involded in cell proliferation and survival, whereas no such effect could be observed on non tumoral cell lines, such as HEK293 cells or primary cells with limited replication (HAEC or human macrophages). What differentiate these cell lines is the unlimited and uncontrolled proliferation capacity of tumor cells which appears to be a requirement for the differential cytotoxic effect of anti-apoA-1 antibodies. Because p53 is the sensor of exogenous and intrinsic cell stresses that potentially induce DNA damage and because mutations of p53 are the most frequently at the origin of uncontrolled proliferation [32], p53 pathway was preferentially investigated. Among the p53 hotspot mutation panel, certain mutations are at the origin of a gain of function. By binding unspecific and promotor-independent sequences distributed widely over the genome, the p53 mutants can induce novel gene transcription and modification of cellular pathways leading to new phenotypes [33, 34]. To this regard, notable difference could be highlighted between U251, SUPT1, Hela and HEK293 cell lines. U251 and SUPT1 hold both an identical p53 mutation (R273H) responsible for their high proliferation rate, whereas HEK293 and primary cells contain wildtype p53 protein [35, 36]. Hela cells present a particular phenotype with a specific repression of p53, due to HPV16 E6 oncoprotein [18, 19, 37], outlining that compensatory mechanisms to p53 may be engaged to induce growth cell arrest and apoptosis in response to anti-apoA-1 autoantibodies in these cells. The fact that p53 is specifically activated via phosphorylation on serine 15, which is sufficient for p53 transcriptional activity [23, 25], cumulated with the up-regulation of VRK2, PLK3 and down regulation of CDKN2AIP genes necessary for p53 stabilisation [38–40] and with the inactivate status of the second central tumor suppressor protein Rb [41], concur to emphasize the central role of p53 in orchestring anti-apoA-1 antibody effects in U251 and SUPT1 tumoral cells. The cell growth arrest in G2/M phase observed in these two cell lines, is compatible with the increased expression of the p53-inducible genes GADD45 alpha, 14.3.3 sigma and CDKN1C, known to block G2/M phase progression by inhibition of the CDK1/Cyclin B complex [42, 43]. The strong decrease of cyclin B protein level and the unchanged phosphorylation status of CDK1 observed in U251 treated with anti-apoA-1 antibodies is in favor of GADD45 alpha implication, as it is known to disrupt CDK1/cyclin B complex inducing cyclin B exportation into the cytoplasm and degradation [20]. In cells lacking functional p53, GADD45 alpha expression can be alternatively induced by MAPK to further mediate cell apoptotis via MEKK4/MTK and Jun/p38 signaling pathway [20]. This mechanism may account for cell growth arrest observed in Hela cell model, but this needs to be further investigated. Other p53-dependent genes identified by our transcriptomic analyses are linked to tumor cell repression and may play an anti-proliferative role on our tumor cell models. Among the strongest expressed ones, it is of interest to note that BTG4 and RERG are known to bare anti-proliferative function in several cancers [30, 31, 44–48], that the peptidase inhibitor P16 inhibits cell proliferation in non cancerous cells [49, 50] and acts as a paralog of GLIPR1 (Glioma Pathogenesis related protein 1), a CAP/CRIPS protein associated with cell growth suppression and proapoptotic activities in multiple cancer cell lines including glioma [51], and finally that SERTAD1 and CDK5R1 were shown to have a specific role in neuronal cell death [52, 53].

**Figure 8:**
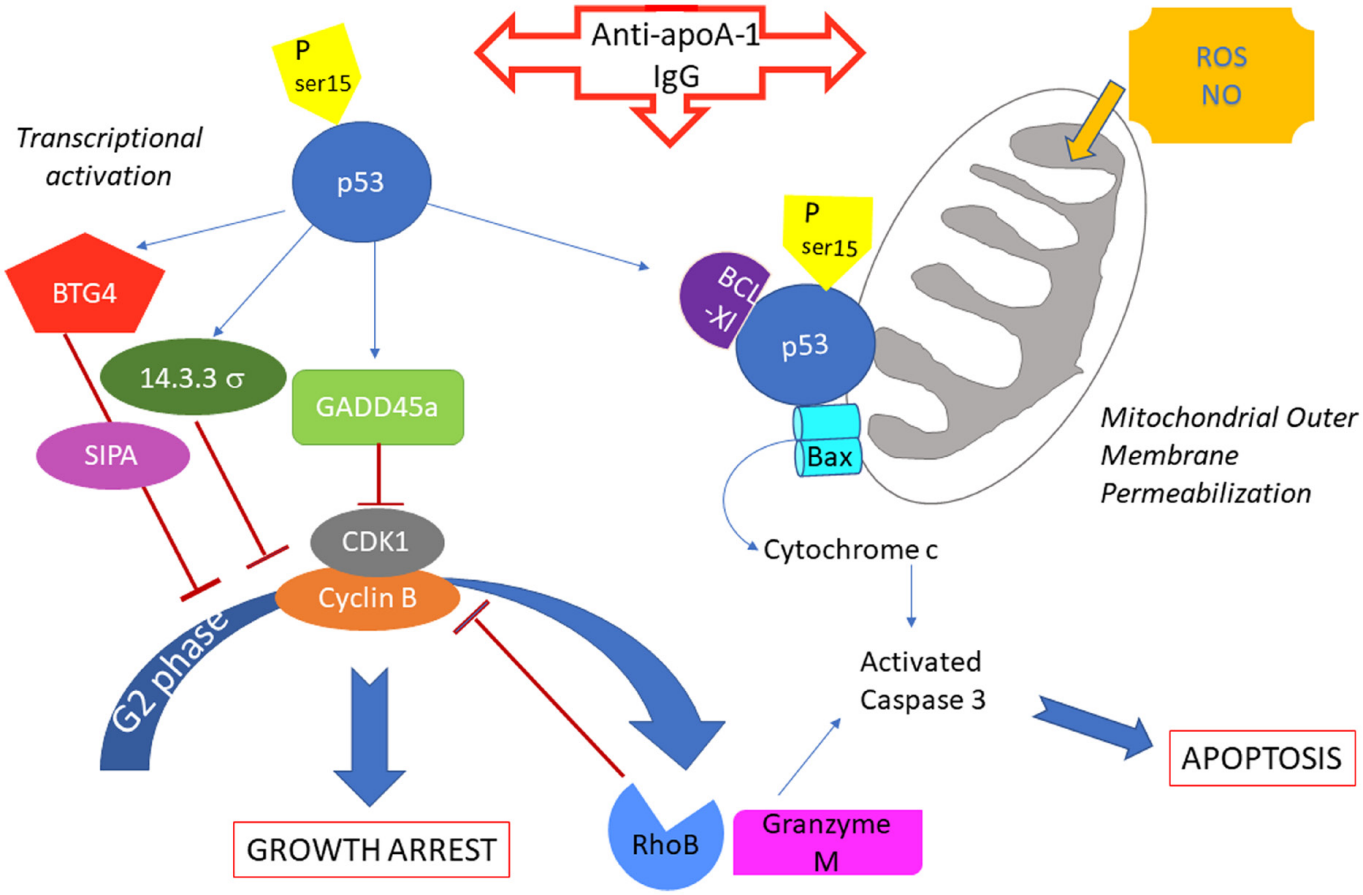
Summary of potential anti-apoA-1 antibody induced signalings involved in cancer cell growth arrest and apoptosis.

In addition, several indications suggest a possible involvement of both extrinsic “death receptor” and intrinsic “mitochondria dependent” apoptotic pathways [54, 55], in the caspase 3-dependent apoptotic effect of anti-apoA-1 antibodies on the tumor cell lines. First, several members of the death receptor family were induced by anti-apoA-1 antibodies. Among them, TNF receptor super family 8 (TNFRSF8 or CD30) and TNFRSF10D (TRAIL Receptor), together with two TNFRSF ligands TNFSF9 (CD137Ligand) and TNFSF4 (Ox40 Ligand) were notably up-regulated and some link to caspase 3-dependent tumor apoptosis have been described for TNFRSF8 (CD30) [56–58] and for TNFSF9 [59, 60], the lastest being also involved in promoting neuronal cell death [61]. TLR6, TLR1, TLR10 and CD14, co-receptors of TLR2 and TLR4 previously identified as anti-apoA-1 antibodies receptors [9, 13], and TLR4 have been transcriptionnaly up-regulated. Although it has been demonstrated that p53 modulated TLR expression in cancer cells [62] and that TLR4 and TLR2 are involved in the apoptosis of neural cells [63, 64], we were previously unable to highlight a direct role of TLR2 and TLR4 in mediating anti-apoA-1 antibody apoptotic effect in U251 and SUPT1 [14, 15]. Secondly, the strong increased expression of NO synthase gene and others involved in ROS generation may indicate the occurance of an important oxidative stress that could be at the origin of a mitochondrial-dependent caspase 3 activation [65, 66]. In addition, although we didn’t find any significant increased expression of the p53-dependent pro-apoptotic genes NOXA, PUMA, BAD, that are the conventional initiators of the mitochondrial outer membrane permeabilization (MOMP) leading to caspase 3 activation [67], a transcriptional independent role for p53 could not be excluded because it has been demonstrated that p53 can directly induce cytochrome c release from mitochondria by forming inhibitory complexes with Bcl-XL and activating Bax through oligomerization triggering MOMP [24, 68, 69]. Finally, Granzyme M and RhoB, also highligthed by RNA sequencing, may be potential actors as they have been shown to contribute to caspase 3 activation in a FADD-caspase 8 dependent or DNA topoisomerase dependent ways, and cell cycle arrest in G2/M phase in neoplastic cells [70, 71]. A summary of our current understanding of the pro-apoptotic effects of anti-apoA-1 IgG on tumoral cells is proposed in Figure 8.

This study presents some limitations. Firstly, by using tumor cells from different genetic background, we intended to capture a clear signaling pathway or a specific tumor target in response to anti-apoA-1 IgGs, but the results indicate that the anti-proliferative capacity of anti-apoA-1 IgGs may be rather generic and probably not restricted to a particular tumor cell type. Nevertheless, further tumoral cell lines testing require to be performed before any formal conclusions can be made. Secondly and along the same line, transcriptomic analysis was performed on the glioma cell line U251 only and by consequent, some gene modifications may concerned neuronal cells and can not be generalized to other neoplastic cells without further investigations, although the principal genes outlined in this study are also common to other cancers [56, 57, 72]. Thirdly, aside p53 and caspase 3, we could not validate the other targets identified by transcriptomic at the protein level. The fourth limitation comes from the fact that U251 and SUPT1 carry the same mutation on p53 (R273H). It would be of interest to investigate whether the deleterious effect of the anti-apoA-1 antibodies occurs on cancerous cell lines with other types of p53 mutation. Indeed, the fact that Hela cells were also sensitive to anti-apoA-1 antibodies, although without functional p53, indicates that other p53-independent mechanisms may also be involved. In that perspective, it would have been interesting to compare the transcriptomic response to anti-apoA-1 antibodies of the different tumorous and non tumorous cell lines to get a clear picture of the common pathways engaged by the antibodies but for a reason of cost, this was not feasible. In addition, all the investigations performed did not allow to identify a receptor or a class of receptors that can be targetted by the anti-apoA-1 antibodies.

At last, the anti-proliferative effects of anti-apo-A1 IgG obtained on tumor cell lines should be analyzed in tumor models to better define for which kind of tumor these antibodies might be valuable and the clinical benefit that can be derived from it. In parallel, as this is a new anti-apoA-1 IgG effect shown using commercial source, it should be of importance to confirm it with antibodies purified from patients, although this was previously the case for all the pathological effects already analyzed.

In conclusion, we report here for the first time that anti-apoA-1 autoantibodies induce cell proliferation arrest and apoptosis in malignant cell lines via activation of p53, down regulation of cyclin B involving GADD45 alpha, and by modulating the inflammatory and oxidative stress response culminating to the activation of caspase 3. Altough further understanding of the other possible mechanisms involved in the apparent specific anti-apoA-1 IgG cytotoxicity on tumoral cells is required, the present results may open new therapeutic perspectives, especially in brain tumors, where an intrathecal infusion of these antibodies could be envisaged in top of other systemic chemotherapy agents. Such hypothesis requires further validation in animal models before any preliminary conclusions can be made.

## MATERIALS AND METHODS

### Cell lines

Human astrocytoma cell line U251 and T cell CD4+ lymphoblastic lymphoma cell line SUPT1 were obtained from Sigma-Aldrich (Schnelldorf, Germany). Human Embryonic Kidney 293A (HEK293A) and adenocarcinoma cervix epithelial HeLa were obtained from ATCC (ATCC-LGC standards GmbH, Wesel, Germany). Human Aortic Endothelial Cells (HAEC) were obtained from Lonza and grew in EGM medium (Lonza, Basel Switzerland). HeLa and HEK293A were cultivated in DMEM medium supplemented with Streptomycin/penicillin antibiotic solution (Gibco BRL-Life Technologies, Grand Island, NY) and with 10% Foetal Bovine serum (FBS) (Gibco BRL). U251 was cultivated in RPMI-1640-GlutaMax culture medium with 1% FBS (Gibco BRL) and SUPT1 in RPMI-1640-GlutaMax culture medium with 10% FBS.

### Monocyte purification

Monocytes were isolated from blood buffy coats of healthy volunteers provided by the Geneva Hospital blood transfusion center, as previously described [9]. Monocyte purity was routinely controlled by flow cytometry and the isolated cell population consisted of CD14^+^ cells (> 90%), CD3^+^ cells (< 1%), and CD19^+^ cells (< 1%). Cells were cultured in RPMI containing 10% FBS.

### Antibodies

Polyclonal goat anti-human apoA-1 IgG (Academy Bio-Medical Compagny, Inc., Houston, TX) (anti-apoA-1 IgG) were used for the *in vitro* studies as surrogates for human anti-ApoA-1 IgG as they were thoroughly validated to have the same specificity and the same pathological activity *in vivo* and *in vitro* as anti-apoA-1-IgG isolated from patients [10, 73, 74]. Polyclonal goat IgG (Ctl IgG)(Meridian Life Science, Saco, ME) were used as isotype control.

### Cell morphology

U251, SUPT1 or HEK293 cells were cultured in 96-well plates in presence of goat polyclonal anti-apoA-1 IgG or isotype CTL IgG at 150 μg/ml for 48 or 72 h. This concentration was previously established by concentration range assays [14, 15]. Pictures of the cultured cells were taken at objective ×10 using a Axiovert 25 (Zeiss) microscope equipped with a CETI SI-3000 High-Definition Digital camera.

### *In vitro* detection of apoptosis and necrosis

Cells were seeded at 2.10^5^ cell/ml in 6 well-plates (Thermo Scientific™Nunc™) and polyclonal goat anti-human apoA-1 IgG or polyclonal goat control IgG were added at 150 μg/ml for 24, 48 or 72 h [14, 15]. For macrophages, the time length is limited to 48 h because of the difficulties to maintain them in regular culture medium for long time.

Necrotic/apoptotic cells were detected using Zombie Red Fixable Viability Kit (BioLegend, San Diego, CA, USA) combined with the PE active caspase-3 apoptosis kit from BD-Pharmingen (San Diego, CA, USA) according to manufacturer protocols. Apoptosis/necrosis was quantified using an Accuri C6 Flow cytometer (BD Biosciences, San Jose, CA, USA). The data were analysed with FlowJo_V10.0.7 software (FlowJo LLC, Ashland, OR, USA). Data are expressed as percentage of cells positive for Zombie dye labelling (necrotic cells) and/or positive for active caspase-3 labeling (apoptotic cells).

### Cell cycle analysis by flow cytometry

Cells were seeded at appropriate concentrations in 12 well plates to be in growth phase at the time of harvest. After 24 h incubation for cell adhesion, goat anti-apoA-1 IgG or goat control IgG were added to the medium at 150 μg/ml and incubated for 24, 48 and 72 h. At each time point, the adherent cells were collected by trypsinization (Trypsin-EDTA 0.05%, Gibco) and washed in D-PBS (Gibco). After centrifugation and supernatant removal, cells were fixed by addition of 500 μl of 70% ice-cold Ethanol dropwise to the pellet while gently vortexing to avoid clumps, followed by 30 min incubation on ice. Cells were then washed once with PBS + 2% FBS and incubated with 100 μl of ribonuclease A at 100 μg/ml (RNAse A, DNAse and Protease free, Thermo Fisher Scientific, MA) for 30 min, followed by the addition of 400 μl of propidium iodide (PI)(Invitrogen Molecular Probes) at 62.5 μg/ml in PBS+2%FBS (final concentration 50 μg/ml) for 30 min.

Sample acquisition was performed using an Accuri C6 Flow cytometer set at slow speed and cell cycle phases analyzed with FlowJo_V10.0.7 software as followed: First, the cell population was gated on a Forward and Scatter dot plot, then this gate was applied on FL2-Area versus FL2-Height dot plot and the single cell population was selected. This single cell gate was applied to a FL2-A (PI) histogram plot with linear axes to get the DNA quantification curve with G0/G1, S and G2/M phases. To quantitate the percentage of cells in each cell cycle phase, the Watson algorithm was used to deconvolute the phases. To optimize the fitting: G1 and G2 phases were constrained, the CV value for G1 phase fitting was adjusted to obtain the lowest Root Mean Square (RMS) Error, which indicates the degree of best fitting. Then the same CV value was applied to the CV of G2 adjustment curve.

### MTT viability assay for cell growth

Cells were seeded at 2.10^4^ cell/100 μl of respective growth medium in a 96 well plate. Polyclonal goat anti-human apoA-1 IgG or polyclonal goat control IgG were added at 150 ug/ml. 1 μM of staurosporine was used as positive control for death induction. Conditions were performed in duplicate. Cell viability was quantified over 4 days using the Cell Titer 96^®^ AQ_ueous_ One Solution cell Proliferation Assay (Promega Corp., Madison, WI). Every 24 h, 20 μl of reagent were added to the 100 μl of cell supernatants and incubated at 37°C, 5% CO_2_. Absorbance was red after 30 min and 1 h of incubation, at 490nm using a FilterMax3 (Molecular Devices, LLC San Jose, CA, USA).

### Phosphorylation and protein analysis by Western blot

Cells were seeded at 1.10^5^ cells in 2 ml of medium in 6 well plates (Thermo Scientific™Nunc™). Polyclonal goat anti-human apoA-1 IgG or polyclonal goat control IgG were added at 150 μg/ml for 24, 48 or 72 h.

At each time points, the cells were washed with cold PBS and lysed on ice with 100 μl of cold lysis buffer (20 mM Tris pH 7.5, 150 mM NaCl, 1%NP-40,0.5% DOC, 0.1% SDS) supplemented with Halt™ Protease and Phosphatase inhibitor cocktail and EDTA (Thermofisher Scientific, Waltham, MA, USA). The lysate was incubated 30 min on ice then sonicated 2 min at 4°C. The lysate was clarified by centrifugation at 12 000 rpm for 20 min at 4°C. Proteins were quantified by Bicinchoninic acid assay (Pierce™, Thermofisher Scientific). Thirty micrograms of proteins were resolved in 10% SDS-Page gel under reducing condition and transferred to nitrocellulose membranes (GE Healthcare Life Sciences, Pittsburgh, PA, USA). Membranes were incubated with the following antibodies from Cell Signaling Technology (Danvers, MA, USA): Phospho-Ser15-p53 mouse monoclonal antibody (#9286), Phospho-Ser46-p53 rabbit antibody (#2521), Phospho-Ser20-p53 rabbit antibody (#9287), p53 monoclonal rabbit antibody (#2527), Cyclin B1 monoclonal rabbit antibody (#12231), Phospho-tyr15 CDK1 monoclonal rabbit antibody (#4539), PARP monoclonal rabbit antibody (#9532), Phospho ser807/811 Rb monoclonal rabbit antibody (#8516), anti-mouse IgG HRP linked antibody (#7076), anti-rabbit IgG HRP-linked antibody (#7074) and anti β-actin mouse monoclonal antibody (ab8226, Abcam). WesternBright Quantum (Advansta) Chemiluminescent substrate and ECL film (Amersham Pharmacia Biotech) were used to reveal the proteins. Relative protein levels were measured using densitometric analysis with Fiji software [75]. Results are expressed as arbitrary unit.

### Transcriptomic profiling by RNAseq

RNA from U251 treated with or without 150 μg/ml polyclonal goat anti-apoA-1 IgG or CTL IgG for 24, 48 and 72 h were purified using Nucleospin RNA isolation kit (Macherey-Nagel, Düren, Germany), including the DNase treatment step. RNA quantification was performed with a spectrophotometer. RNA seq experiment was performed at the *IGE3* Genomics Plateform of the University of Geneva (https://ige3.genomics.unige.ch). RNA integrity was assessed with an Agilent 2100 bioanalyser (Agilent Technologies, Inc), all samples presented a RNA Integrity number (RIN) greater than 7.9. Samples were proceeded in duplicata. Library preparation was performed from stranded mRNA with the *Illumina TruSeq* protocol and 50 paired-end sequencing was completed on a HiSeq 4000. The sequencing quality control was done with FastQCv.0.11.5. The reads were mapped with the STARv2.5.3a software to the reference genome *Homo sapiens* –UCSC-hg38. The normalization and differential expression analysis was performed with the R/Bioconductor package edgeR v.3.16.5, for the genes annotated in the reference genome. Approximatively fifty millions reads per sample were generated, with an average percentage of uniquely mapped reads to *Homo sapiens* genome (UCSC hg38) of 86%, and with 57% of aligned reads assigned to a gene. The number of genes differentially expressed by comparing anti-apoA-1 IgG (aAPO) condition with Ctl IgG or with untreated cell (Cell) conditions for each time point is resumed in Supplementary Table 1.

Pairwise comparisons (General linear model, quasi-likelihood F-test) were performed between the different conditions at each timing. Differential gene expressions were extracted with the following thresholds: *p*-value ≤ 0.05 (FDR with Benjamini-Hochberg correction for multiple testing) and fold-change value ≥ 2. Venn Diagram were used to identify genes that are specifically expressed in cell treated with anti-apoA-1 IgG versus untreated and cells treated with Ctl IgG (see Supplementary Figure 1). To identify genes implicated in apoptosis and cell cycle, we cross-mapped the gene lists with the apoptosis, DNA damage and cell cycle pathway maps in MetaCore (GeneGo, Inc., MI).

## SUPPLEMENTARY MATERIALS




