# Supplementary material for: Auto-antibodies against apolipoprotein A-1 block cancer cells proliferation and induce apoptosis
Source: Oncotarget. 2020 Nov 17;11(46):4266–80. doi: 10.18632/oncotarget.27814 (PMC7679029; doi:10.18632/oncotarget.27814)
Supplement: Supplementary file 1 [file oncotarget-11-4266-s001.pdf]

Auto-antibodies against apolipoprotein A-1 block cancer cells proliferation and induce apoptosis

SUPPLEMENTARY MATERIALS

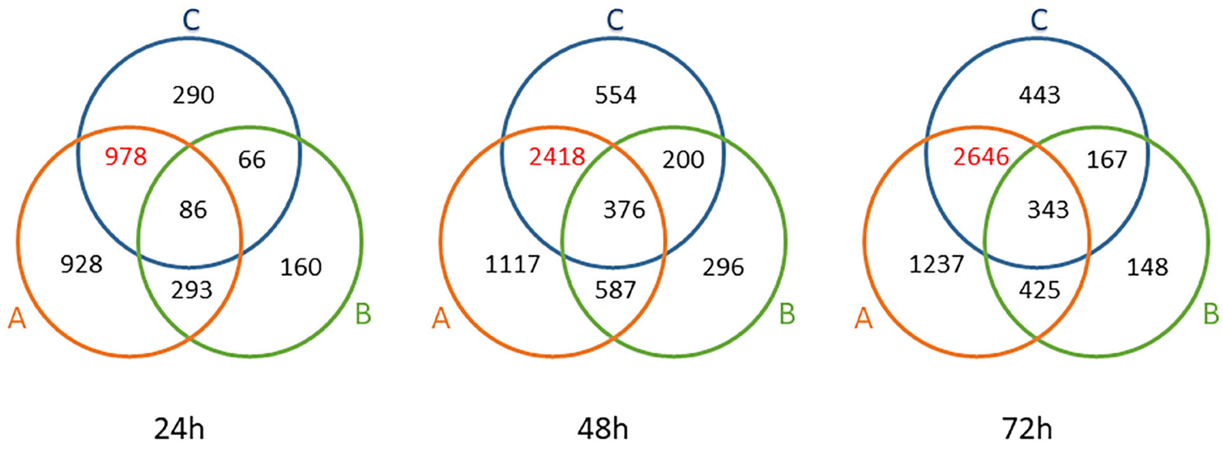

Supplementary Figure 1: Differential expressed genes across cell treatment comparisons. The lists of differentially expressed genes obtained by pairwise comparisons using raw *p*-value < 0.05 and Fold change > 2, were plotted in Venn diagrams. (**A**) significant genes expressed by anti-apoA-1 IgG treated cells (aAPO) versus untreated cell (Cell), (**B**) significant genes expressed by Ctl IgG treated cells (Ctl IgG) versus untreated cell (Cell), (**C**) significant genes expressed by aAPO versus Ctl IgG. The genes that are specific to anti-apoA-1 IgG treatment but are not found either in Cells or Ctl IgG conditions are in the intersection of A and C but not B circle (number in red).

Supplementary Table 1: Number of significant genes differentially expressed by comparison

Comparison	Nb of significant genes (at 5% raw *p*-value & FC>2)	Nb of significant genes (at 5%FDR* & FC>2)
aAPO versus cell 24 h	2 285	2 203
aAPO versus cell 48 h	4 498	4 448
aAPO versus cell 72 h	4 498	4 615
Ctl IgG versus cell 24 h	605	465
Ctl IgG versus cell 48 h	1 459	1 332
Ctl IgG versus cell 72 h	1 083	947
aAPO versus Ctl IgG 24 h	1 420	1 309
aAPO versus Ctl IgG 48 h	3 548	3 495
aAPO versus Ctl IgG 72 h	3 599	3 547

*FDR: False Discovery Rate (Benjamini & Hochberg) correction for multiple testing. FC: fold change.

Supplementary Table 2: Groups of genes significantly up or down-regulated by apoA-1 IgG and linked to apoptosis and cell cycle pathways. See Supplementary Table 2
