# Supplementary material for: Auto-antibodies against apolipoprotein A-1 block cancer cells proliferation and induce apoptosis
Source: Oncotarget. 2020 Nov 17;11(46):4266–80. doi: 10.18632/oncotarget.27814 (PMC7679029; doi:10.18632/oncotarget.27814)
Supplement: Supplementary file 2 [file oncotarget-11-4266-s002.docx]

**Supplementary Table 2: Groups of genes significantly up or down-regulated by apoA-1 IgG and linked to apoptosis and cell cycle pathways**

| **Groups** | **Gene ID** | **Protein ID^a^** | **FC^b^**  **24h** | **FC 48h** | **FC 72h** | **Function^a^** |
| --- | --- | --- | --- | --- | --- | --- |
| **Cell cycle and cell growth** | SFN | 14.3.3 sigma | −2.27 | 3.25 | 3.08 | p53-regulated inhibitor of G2/M progression- inhibitor of CDC25C and CDK1 |
|  | GADD45A | Growth arrest and DNA damage inducible protein alpha | — | 5.35 | 9.91 | Cell cycle arrest and apoptosis process. Inhibition of CDK1/cyclin B |
|  | GADD45B | Growth arrest and DNA damage inducible protein beta | — | 4.15 | 6.07 | Cell cycle arrest and apoptosis process. mediates activation of stress responsive MTK1/MEKK4 MAPKKK |
|  | GADD45G | Growth arrest and DNA damage inducible protein gamma | — | 4.34 | 2.73 | Involved in the regulation of growth and apoptosis. Mediates activation of stress-responsive MTK1/MEKK4 MAPKKK. |
|  | CDKN1C | cyclin-dependent kinase inhibitor 1C- P57 ^kip2^ | 2.17 | 16.9 | 7.97 | Inhibitor of cell proliferation, binding inhibitor of Cyclin B/CDK1 |
|  | CDKL2 | Cyclin-dependent kinase like 2/p65 | — | 7.67 | 6.82 | Discrepancy role in cell cycle regulation |
|  | CDKN2D | Cyclin dependent kinase 4 inhibitor D | 3.46 | 3.96 | 2.94 | INK4 family of cyclin-dependent kinase inhibitors-inhibit CDk4/6 and control G1 progression |
|  | CDK5R1 | Cyclin-dependent kinase 5 activator 1: p35 | 2.4 | 4.46 | 3.24 | Activator of CDK5 that mediates apoptosis in glioblastoma (ref 51) |
|  | SIPA1 | Signal-induced proliferation-associated protein 1 (SPA-1) or GTPase activating protein (GAP) | 6.9 | 15.13 | 11.11 | Affect cell cycle progression, Activate Ras-related regulatory protein Rap1 and Rap2, hamper mitogen-induced cell cycle progression |
|  | SERTAD1 | Serta domain containing protein 1 (p34-SE-1) | — | 5.74 | 6.26 | Cell cycle regulatory protein, direct activator of cdk4 and p53, involved in neuron cell death (ref 50) |
|  | FOSL1 | Fos-related Antigen 1 | 2.68 | 5.3 | 9.46 | transcription activator, negative regulation of cell proliferation, positive regulation of apoptosis |
|  | PI16 | peptidase inhibitor 16 | — | 78.98 | 45.45 | Negative regulation of cell growth |
|  | PLK2 | Serine/threonine protein kinase | — | 2.59 | 3.59 | Its induction by p53 suggests that it may participate in the mitotic checkpoint following stress |
|  | RERG | Ras-related and estrogen-regulated growth inhibitor | 29.18 | 328.42 | 51.82 | Inhibit cell proliferation and tumor formation, involved in ras/Mek/ERK pathway; high expression reduces proliferation rate |
|  | BTG4 | BTG antiproliferation factor 4 (other name: PC3b) | — | 129.8 | 319.11 | Cell cycle arrest, negative regulation of mitotic cell cycle |
|  | CAMK1 | Ca/calmodulin dependent protein kinase 1 | — | −4.73 | −5.13 | Requires for proper activation of cyclin D1/CDK4 complex during G1 phase |
|  | TP53I3 | P53 inducible gene 3 (PIG3) Quinone oxidoreductase | −3.13 | −5.36 | −3.48 | Involves in DNA damage repair. Its down expression induces G2/M arrest. (ref 31) |
| **P53 stabilisation and regulation** | VRK2 | seronine/threonine protein kinase VRK2 | — | 445.88 | — | Reduces p53 ubiquitination by MDM2; promotes p53 acetylation by EP300 and thereby increases p53 stability and activity |
|  | PLK3 | Serine/thréonine protein kinase | — | — | 4.12 | Phosphorylates p53 in response to reactive oxygen species (ROS), thereby promoting p53 mediated apoptosis |
|  | CDKN2AIP | CARF: CDKN2A interacting protein | −3.53 | −7.81 | −4.62 | Inverse regulator of p53 (ref 37, 38) |
| **Apoptosis regulation** | BIRC3 | cIAP-2 | 3.21 | 48.92 | — | Regulator of caspase and apoptosis |
|  | RHOB | Rho-related GTP binding protein Rho b | — | 8.55 | 4.81 | Mediator of apoptosis in neoplastic cells (ref 69) |
|  | PMAIP1 | NOXA | — | — | 3.53 | Promote activation of caspases and apoptosis |
|  | GZMM | Granzyme M | — | — | 9.46 | Activator of caspases, cleaves BCR5/survivin |
|  | NDRG1 | NDRG1 protein | 4.2 | 3.08 | 2.13 | Pro-apoptotic gene; stress response protein |
|  | PAK1 | Serine/threonine protein kinase PAK1 | — | −4.3 | −3.31 | Reduction of PAK1 activity initiates caspase 3 activation. |
|  | TP53AIP1 | Tumor protein p53-regulated apoptosis-inducing protein 1 | — | 5.32 | — | Tumor suppressor gene-Regulates mitochondria permeability and cytochrome C release |
|  | TNFRSF10D | TRAIL receptor/TRAIL R4 | 24.54 | 227 | 288 | regulator of apoptosis, NF-KB activation |
|  | TNFRSF10C | TRAIL receptor/TRAIL R3 | — | −5.69 | −4.8 | Regulator of apoptosis, NF-KB activation |
|  | TNFRSF8 | TNF receptor super family member 8- CD30 | — | 77.54 | 24.05 | Biomarker of malignancy Positive regulator of apoptosis, NF-kB activation (ref 54, 55) |
|  | TNFRSF19 | TNF receptor super family member 19 | — | −3.06 | −7.34 | Activation of JNK and NF-kB, promote caspase independent apoptosis |
|  | TNFSF9 | TNF super family ligand 9 (CD137L) | — | 129.83 | 75.85 | Immune check point Role in activation-induced cell death |
|  | TNFSF4 | Ox40 Ligand | — | 5.53 | 5.22 | Immune check point protein |
|  | TRAF5 |  | — | −2.73 | — | Adaptor of TNFR family, associates with TNFRFS8, TNFRFS19, mediates activation of NF-kB and JNK |
|  | TRAF3 (AMN) | TRAF3 | — | 12.95 | 22.31 | Adaptor of TLR family, involved in INF production and in NF-KB2 associated apoptosis |
|  | PAK1 | serine/threonine protein kinase | — | −4.1 | −3.32 | actin cytosqueleton dynamic, and apoptosis process. Activity is inhibited in cells undergoing apoptosis |
|  | JAK2 | tyrosine protein kinase JAK2 | — | −2.28 | −2.85 | Activation of cell proliferation via MAP3K Inhibition of apoptosis via PI3K/AKT |
|  | IRAK1BP1 | IRAK1 binding protein 1 | — | −4.13 | −3.58 | IRAK1-dependent TNFRSF1A signaling pathway component Required for cell survival |
| **Inflammation regulation** | TLR4 | TLR4 | — | — | 4.9 | Death receptor family, Toll-like receptor 4 |
|  | TLR6 | TLR6 | — | 6.12 | 3.13 | Death receptor family, coreceptor of TLR2 |
|  | TLR1 | TLR1 | — | — | 2.44 | Death receptor family, coreceptor of TLR2 |
|  | TLR10 | TLR10 | — | 18.37 | — | Death receptor family, coreceptor of TLR2 |
|  | CD14 | CD14 | — | 3.66 | 4.06 | Cofactor of TLR2 and TLR4 |
|  | CXCL11 | I-TAC/CXCL11 | — | 72.36 | 61.5 | Inhibition of tumor growth via CXC3 receptor |
|  | CXCL10 | IP10/CXCL10 | — | 9.24 | 9.84 | Regulation of cell growth and apoptosis inductor |
|  | PRKCZ | PKC-zeta | −2.5 | −6.46 | −4.31 | MAPK, NF-kB activator |
|  | NFKB2 | p100/p52 | 2.18 | 9.48 | 8.66 | Non-canonical NF-kB pathway, transcriptional factor |
|  | NFATC1 | Ik-b | — | −3.03 | −3.46 | Inhibitor of NF-kB |
|  | GNG7 | Guanidine nucleotide binding protein subunit gamma | −2.15 | −10.19 | −20.96 | Requires for GTPase activity ; Regulation of adenylyl cyclase activity |
|  | MAP2K3 | MEK3 | — | 3.19 | 3.92 | Phosphorylation of p38 |
|  | MAPK4 | MAPK4 (ERK-4) | — | — | −4.38 | May promote entry in the cell cycle |
|  | MAPK13 | P38 MAPK | — | 3.34 | — |  |
|  | JUN | tAP-1 | — | 2.64 | 3.1 | Transcription factor |
| **Oxidative stress** | NOS3 | Nitric oxyde synthase | — | 125.74 | 349.5 | Production of NO. downregulation of cell proliferation |
|  | DHRS2 | Dehydrogenase/reductase SDR family member 2 | — | 57.14 | — |  |
|  | RAC2 | ras-related protein Rac | — | — | 15.95 | increase production of ROS by NADPH oxidase |
|  | CAMK2A | Ca/calmodulin dependent protein kinase II (CAMKII) | 3.8 | 26.07 | 19.73 | Enhance NADPH oxidase 5 (Nox5) activity and extracellular ROS production |
|  | NOXA1 | NADPH oxidase 1 activator protein | — | −7.38 | −6.95 | Activator of the NADPH oxidase 1 |
|  | TP53I3 | P53 inducible gene | −3.13 | −5.36 | −3.48 | production of ROS, apoptosis inductor |

^a^ source of information from uniport. org or from pubmed literature.

^b^FC: Fold Change (p-value ≤ 0.05 with Benjamini-Hochberg correction for multiple testing).
